# Synthesis of a PVA drug delivery system for controlled release of a Tramadol–Dexketoprofen combination

**DOI:** 10.1007/s10856-021-06529-3

**Published:** 2021-05-07

**Authors:** Juan Carlos Flores-Arriaga, Daniel Chavarría-Bolaños, Amaury de Jesús Pozos-Guillén, Vladimir Alonso Escobar-Barrios, Bernardino Isaac Cerda-Cristerna

**Affiliations:** 1 Advanced Polymers Lab, Instituto Potosino de Investigación Científica y Tecnológica (IPICYT), Camino a la Presa 2055, Lomas 4a, 78216 San Luis Potosí, SLP Mexico; 2 Diagnostic and Surgical Sciences Department, Faculty of Dentistry, Universidad de Costa Rica, San Jose, Costa Rica; 3 Basic Science Laboratory, Faculty of Dentistry, San Luis Potosi University, Manuel Nava 2, Zona Universitaria, 78290 San Luis Potosí, SLP México; 4 Facultad de Odontología, Región Orizaba-Córdoba, Universidad Veracruzana, Abasolo Sur, SN, Tenango de Río Blanco, 94732 Veracruz, México

## Abstract

The local administration of analgesic combinations by means of degradable polymeric drug delivery systems is an alternative for the management of postoperative pain. We formulated a Tramadol–Dexketoprofen combination (TDC) loaded in poly(vinyl alcohol) (PVA) film. Films were prepared by the solvent casting method using three different molecular weights of PVA and crosslinking those films with citric acid, with the objective of controlling the drug release rate, which was evaluated by UV–vis spectrometry. Non-crosslinked PVA films were also evaluated in the experiments. Differential scanning calorimetry (DSC) analysis of samples corroborated the crosslinking of PVA by the citric acid. Blank and loaded PVA films were tested in vitro for its impact on blood coagulation prothrombin time (PT) and partial thromboplastin time (PTT). The swelling capacity was also evaluated. Crosslinked PVA films of higher-molecular weight showed a prolonged release rate compared with that of the lower-molecular-weight films tested. Non-crosslinked PVA films released 11–14% of TDC. Crosslinked PVA films released 80% of the TDC loaded (*p* < 0.05). This suggests that crosslinking films can modify the drug release rate. The blank and loaded PVA films induced PT and PTT in the normal range. The results showed that the polymeric films evaluated here have the appropriate properties to allow films to be placed directly on surgical wounds and have the capacity for controlled drug release to promote local analgesia for the control of postoperative pain.

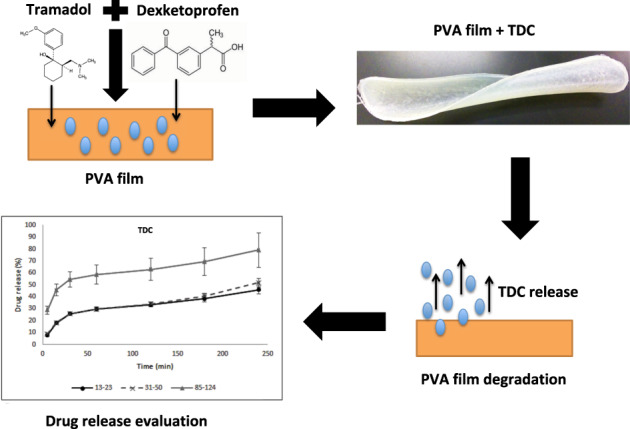

## Introduction

Postoperative pain (PP) management is critical for successful treatment after a surgical procedure. The International Association for the Study of Pain defined PP as a clinical discomfort that can last more than 2 months after surgery [1]. Oral analgesics are the most common pharmacological option to treat PP. Some of the main drugs available for treating PP are central antipyretic/analgesics (paracetamol), nonsteroidal anti-inflammatory drugs (NSAIDs), opioids (codeine, morphine), dual analgesics (Tramadol, Tapentadol), local anesthetics, analgesic coadjutants, or combinations of these [2, 3].

When pain intensity is classified as moderate to severe, the reduction of pain is difficult to achieve with only a single analgesic drug. The combination of analgesic drugs is recommended for those cases by using drugs with different pharmacodynamics profiles to increase the analgesic effect; several analgesic combinations in low doses have been tested for the management of PP [4–6]. An efficient combination of analgesic drugs depends on the correct selection of each drug as well as the correct proportions and doses. The combination should enhance analgesic efficacy and safety compared with those of single drugs [7].

Dexketoprofen is an anti-inflammatory and analgesic drug capable of inhibiting cyclooxygenase pathways (COX1 and COX2). It is effective for pain relief in the acute symptomatic period and has shown benefits in a wide range of clinical conditions [8]. Tramadol hydrochloride is an analgesic drug with a dual-mode of action, combining opioid and non-opioid mechanisms which are thought to act synergistically on descending inhibitory pathways in the central nervous system, resulting in the modulation of second-order neurons in the spinal cord [7]. Although Dexketoprofen and Tramadol are routinely administered systemically, NSAIDs and Tramadol offer valuable evidence in support of a direct local analgesic effect, favoring the possibility for peripheral pain control [7]. Three mechanisms of action contribute to the multimodal analgesia of the Tramadol–Dexketoprofen combination (TDC): the analgesic and anti-inflammatory action of Dexketoprofen, opioid receptor activation by Tramadol, and the indirect activation of central descending mono-aminergic pathways, thus inhibiting nociceptive signal transmission [7]. Studies have shown the effective use of TDC to promote analgesia after surgery by oral and local administration, and effective analgesia and a synergistic effect of TDC have been observed to control PP. Local drug release allows for the administered doses to be decreased, thus reducing potential adverse effects such as vomiting, nausea, gastrointestinal illness, and dependency [7, 9, 10].

Polymers are attractive biomaterials for the formulation of drug delivery systems promoting the release of a combination of drugs. Poly(vinyl alcohol) (PVA) is a biocompatible, biodegradable hydrophilic synthetic polymer with adequate properties to serve as a film for drug delivery [11]. Moreover, PVA has shown non-immunogenic and non-carcinogenic properties [12]. PVA is a polymer suitable for the formulation of a film loaded with TDC to promote a sustained local release of those analgesic drugs, since, by secondary interaction such as hydrogen bridges, the hydroxyl groups pending from the backbone could interact with functional groups from analgesic drugs. Local analgesia is accepted and applied in many clinical approaches. To extend its analgesic effect beyond intervention is critical and, in some cases, difficult [13]. Rapid absorption and biodistribution of local drugs limit their clinical effect, and a second intervention such as systemic medication is routinely needed. Hence, a PVA drug delivery system loaded with TDC might help to solve those problems.

The aim of this study was to synthesize, characterize, and evaluate PVA films loaded with TDC for the local release of TDC. We considered that PVA could establish secondary interaction with functional groups of TDC, helping to retain them. However, it is also well-known that such secondary interactions can create solvation spheres by water molecules, dissolving the polymeric chains. Thus, we decided to evaluate the effect of crosslinking of PVA, using citric acid, on the drug release rate. First, we synthesized PVA films using citric acid as a crosslinker and then loaded them with TDC; PVA films loaded with TDC without citric acid were also prepared. Second, PVA-based films were scanned by scanning electron microscopy (SEM). Third, we evaluated in vitro the release of TDC on films with and without citric acid, as well as the effect of the crosslinker on kinetic release. The effects of the PVA films on blood coagulation were evaluated in vitro.

## Materials and methods

### Drugs and reagents

PVA of three different molecular weights was used (13,000–23,000, 98% hydrolyzed; 31,000–50,000, 98–99% hydrolyzed; and 85,000–124,000, 99+% hydrolyzed) (Sigma-Aldrich, St. Louis, MO, USA) without any modifications. Tramadol HCl and Dexketoprofen trometamol were provided by Stein Laboratories S.A. (Cartago, Costa Rica) and used without excipients or vehicles. Reactive-grade citric acid anhydride (Karal S.A. de C.V. León Gto., México) was used as a crosslinking agent.

### Synthesis of PVA films loaded with TDC

Two different batches were prepared as follows. For the first batch, aqueous solutions of three different molecular weights of PVA were prepared with 10% w/w concentration, with magnetic stirring at 120 rpm/60 °C. When the PVA was completely dissolved, TDC was added slowly to the solution at a proportion of 1.48:1 w/w Tramadol:Dexketoprofen according to Isordia-Espinoza et al. [4]; stirring and temperature were maintained to achieve a homogeneous mixture. For our experiments, 37 mg/ml Tramadol and 25 mg/ml Dexketoprofen were mixed to achieve the ratio of both drugs necessary for TDC. The resulting mixtures were poured into polytetrafluorethylene (PTFE) plates and stored in a drying oven at 50 °C for 24 h to complete the dehydration process. The obtained materials were stored in 50-ml conical tubes, sealed, and protected from light until use.

The second batch was prepared following the same procedure but with the addition of citric acid as a crosslinker at 0.05% w/w with respect to PVA. The resulting mixtures were poured into PTFE plates (15 cm long, 2 cm wide, and 2 mm thick) and stored in a drying oven at 50 °C for 24 h. The temperature was then raised to 160 °C and maintained for 30 min to enhance the crosslinking esterification reaction between citric acid and the OH groups of PVA. We considered that the temperature of 160 °C applied to films to perform the crosslinking would not affect the drug molecules, since, in this stage of synthesis, the films were completely dry, solid, and semi-flexible. The obtained materials were stored in sealed 50-ml conical tubes and protected from light until use (Table 1).

**Table 1:** PVA films codes

Sample name	PVA Mw	Citric acid 0.05%
13–23	13,000–23,000	No
13–23 cx	13,000–23,000	Yes
31–50	31,000–50,000	No
31–50 cx	31,000–50,000	Yes
85–124	85,000–124,000	No
85–124 cx	85,000–124,000	Yes

### SEM analysis

Images were obtained in a FEI-Quanta 200 SEM unit (Thermo Fisher Scientific, Waltham, MA, USA). Samples were collected and sputter-coated with gold. Microscopic images of three different areas in the samples were registered.

### Thermal characterization

Thermal properties of the different samples, whether crosslinked or not, were determined by means of a TA Instrument model DSC Q2000 differential scanning calorimeter. Thus, 10 ± 1 mg of each sample was placed in the aluminum sample holder. A nitrogen atmosphere was used during the analysis. First, it was stabilized at 30 °C, isothermal for 1 min, and then heated up to 200 °C at a rate of 20 °C/min, to erase the thermal history of the sample. Subsequently, it was cooled to 30 °C, isothermal for 1 min, and then heated up to 200 °C at a rate of 10 °C/min.

### Fourier transform infrared spectroscopy

The identification of the chemical species in the samples with and without crosslinking, loaded with TDC, was performed with the Thermo Scientific brand Nicolet iS10 Fourier Transform Infrared Spectrophotometer, in the attenuated total reflectance (ATR) mode. The spectra were recorded at room temperature in the wavenumber range from 500 to 4000 cm^−1^, with 64 scans and a resolution of 4 cm^−1^ for each sample.

### Evaluation of swelling capacity

Swelling capacity was evaluated as follows: a 20-mg quantity of each PVA film loaded with TDC was placed in 15-ml conical tubes. Five millimeters of deionized water were added to each tube, which was then placed in an incubation shaker at 37 °C and 60 rpm. We evaluated the swelling behavior every 60 min by taking the PVA films out of the tubes and blotting them on filter paper to remove excess water, then weighing each film. The time at which the film’s weight stopped changing was registered, indicating equilibrium swelling (*Te*). The experiment was stopped when the films showed physical dissolution or disintegration. All experiments were done in triplicate. The swelling capacity was calculated as follows:

$${\rm{Swelling}}\,{\rm{capacity}}\,\left( {\% W} \right) = \left( {Ws-Wd/Wd} \right) \times 100$$

where *Wd* is the weight of the dry film and *Ws* is the weight after swelling.

### In vitro drug release study

Drug release was evaluated by UV–Vis spectrometry (VARIAN 50 Bio, Santa Clara, CA, USA) at two different wavelengths: 260 nm and 271 nm for Dexketoprofen and Tramadol, respectively. A portion of each of three different molecular-weight PVA films corresponding to ~2 mg of loaded TDC was fitted into a dialysis membrane (average flat width, 10 mm) (Sigma-Aldrich, St. Louis, MO, USA) with 1 ml of deionized water (pH 7.0) and placed with 5 ml of deionized water into test tubes in an incubation shaker at 37 °C and 60 rpm. Aliquots of 3 ml were withdrawn from the test tubes and placed on a quartz cell at 5-min intervals for the measurement of absorbance, and the solution was replaced with equivalent amounts of fresh dissolution media. Experiments with blank samples were performed simultaneously. Experiments were done in triplicate. The percent cumulative concentration of TDC released was calculated to compare the different formulations of PVA film. Two-way ANOVA was used to compare total release of TDC, and the Kruskal–Wallis ANOVA was used to compare between and among groups. A significance level of *p* < 0.05 was established.

### Evaluation of blood coagulation: prothrombin time (PT) and partial thromboplastin time (PTT)

The Ethics Committee of the School of Dentistry of the Universidad Veracruzana approved the experiment. The evaluation of PT and PTT was performed according to a methodology reported in previous studies performed for our research group [14, 15]. For this experiment, the samples were coded as follows: A1-Blank PVA film Mw 13,000–23,000, A2-PVA film Mw 13,000–23,000 loaded with TDC without citric acid, A3-PVA film Mw 13,000–23,000 loaded with TDC and crosslinked with citric acid; B1-Blank PVA film Mw 31,000–50,000, B2-PVA film Mw 31,000–50,000 loaded with TDC without citric acid, B3-PVA film Mw 31,000–50,000 loaded with TDC and crosslinked with citric acid; and C1-Blank PVA film Mw 85,000–124,000, C2-PVA film Mw 85,000–124,000 loaded with TDC without citric acid, C3-PVA film Mw 85,000–124,000 loaded with TDC and crosslinked with citric acid. We collected human blood from a healthy male adult donor. The blood was collected in blood collection tubes with ethylenediaminetetraacetic acid as the anticoagulant. For samples A1, A3, B1, B3, C1, and C3, we took 5 mg of each PVA film and placed it into 1.5-ml conical tubes containing 250 ml of whole blood, after which we incubated the tubes at 37 °C in an incubator shaker (50 rpm, MaxQ 4000 Benchtop Orbital Shaker, Thermo Fisher) for 15 min. After incubation, the samples were centrifuged for 5 min at 3000 rpm to separate the plasma from the cell content. Immediately thereafter, the plasma was used to measure the PT and PTT. For samples A2, B2, and C2, we followed the same experimental protocol, but the samples were incubated with plasma (250 ml) because they caused hemolysis, and the plasma sample showing hemolysis was not suitable for the PT and PTT assays. Each PVA film was tested in triplicate. The PT and PTT assays were performed in a Coagulometer BioBas 10 with its corresponding reagents (Spin React, Girona, Spain); the assay was performed according to the instructions of the manufacturer. Results were analyzed by Kruskal–Wallis ANOVA, Dunn’s post hoc test, and the Mann–Whitney *U* test.

## Results

### SEM analysis

**Fig. 1:**
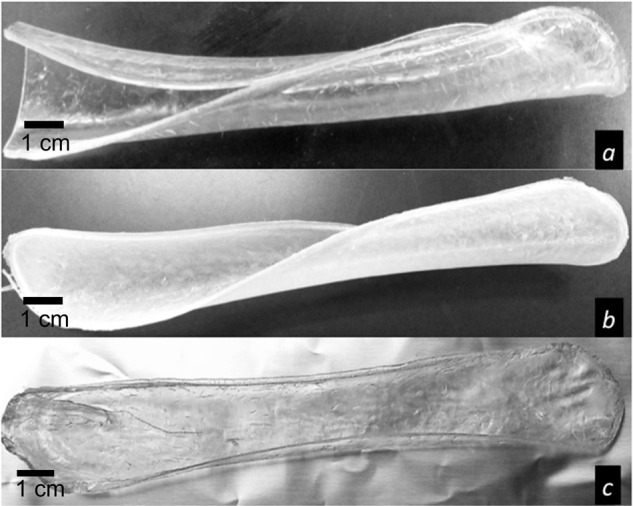
Macroscopic appearance of the PVA-based films: **a** PVA film not crosslinked and not loaded with TDC; **b** PVA film not crosslinked and loaded with TDC; and **c** PVA film crosslinked with citric acid and loaded with TDC

**Fig. 2:**
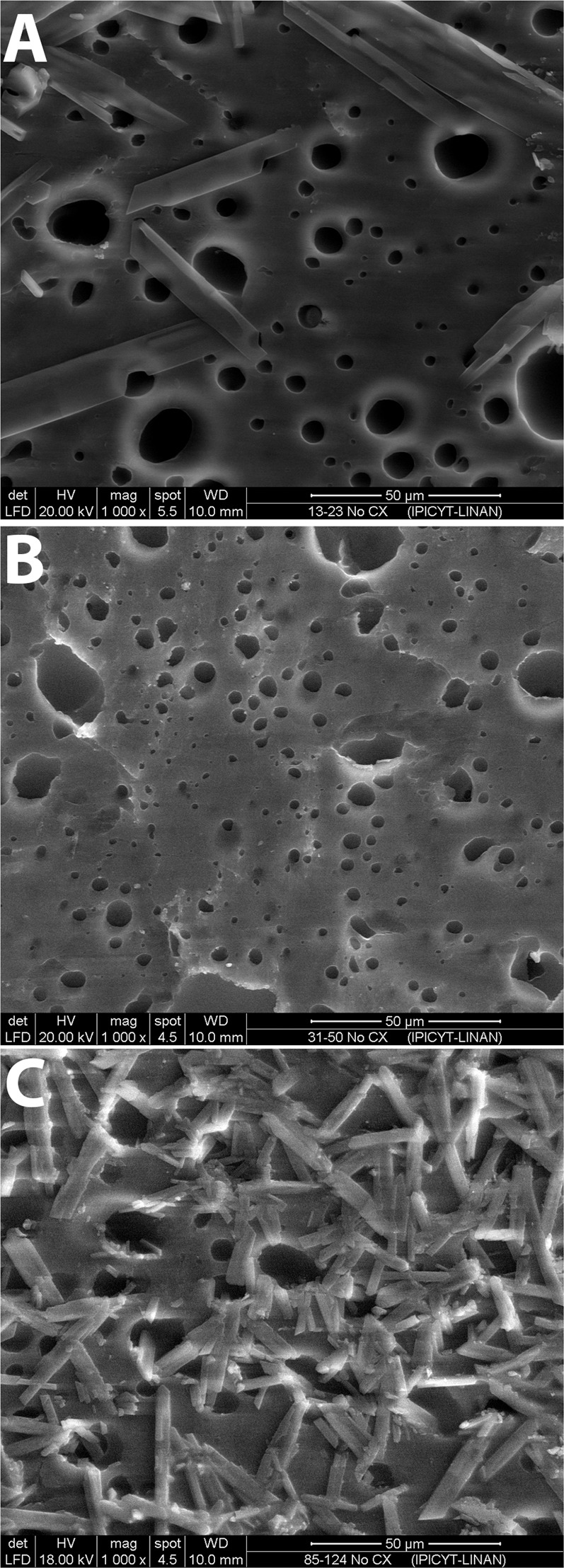
Scanning electron microscopy of non-crosslinked PVA-TDC films: (**A**) 13–23, (**B**) 31–50, and (**C**) 85–124

**Fig. 3:**
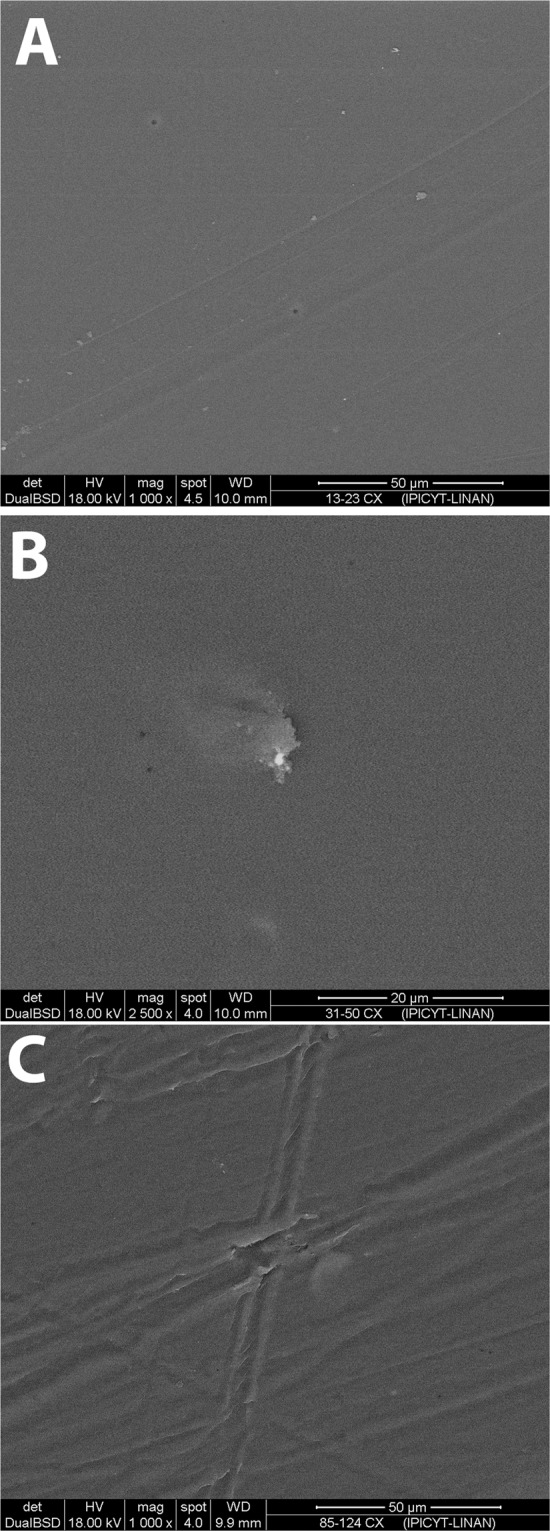
Scanning electron microscopy of crosslinked PVA-TDC films: **A** 13–23 cx, **B** 31–50 cx, and **C** 85–124 cx

Figure 1 shows a macroscopic view of the PVA films. SEM analysis showed two different surface patterns. Figure 2 shows non-crosslinked films, while Fig. 3 shows the films crosslinked with citric acid. Non-crosslinked films exhibit the presence of multiple irregular porous and polygonal structures distributed over the entire surface. Crosslinked films show a homogeneous nonporous smooth surface, with no identifiable polygonal structures.

### Thermal characterization

**Fig. 4:**
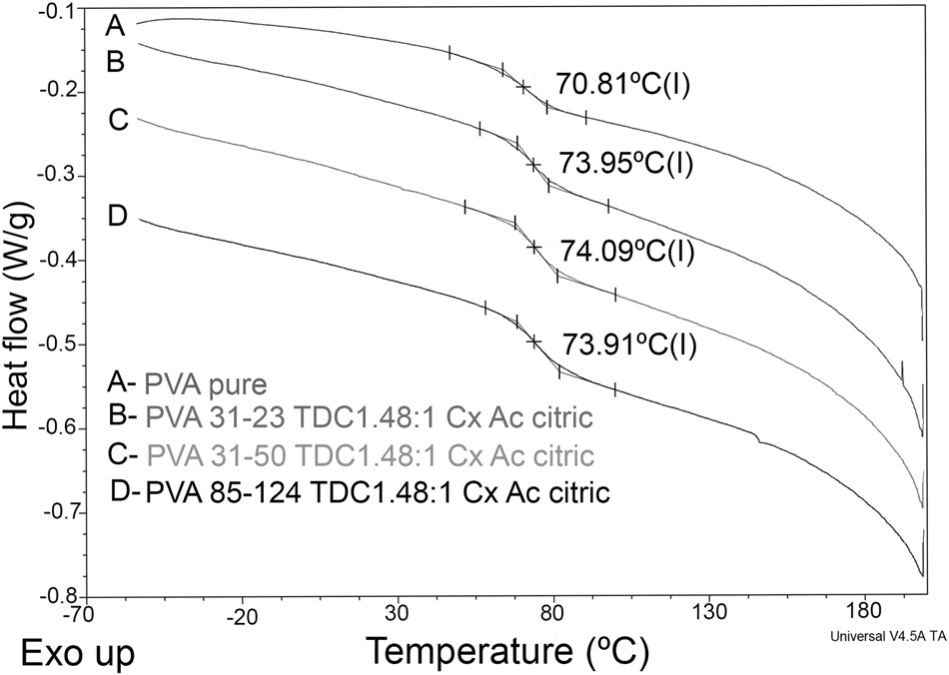
Thermograms of the raw materials, PVA, Dexketoprofen, and Tramadol, as well as the thermograms of crosslinked samples

Thermal analysis of the samples showed differences when they were crosslinked, increasing the glass transition temperature (Tg) signal. Figure 4 shows the thermogram for PVA as well as the thermograms of crosslinked samples. While the PVA showed a clear Tg at 70.81 °C for non-crosslinked samples, the crosslinked samples showed a higher Tg value around 74 °C for each sample. In addition, the samples first loaded with TDC and then evaluated for their release did not show the melting transition corresponding to Dexketoprofen and Tramadol, which is indicative of the non-presence of such molecules; i.e., the TDC was released from the crosslinked PVA matrix.

### Fourier transform infrared spectroscopy

**Fig. 5:**
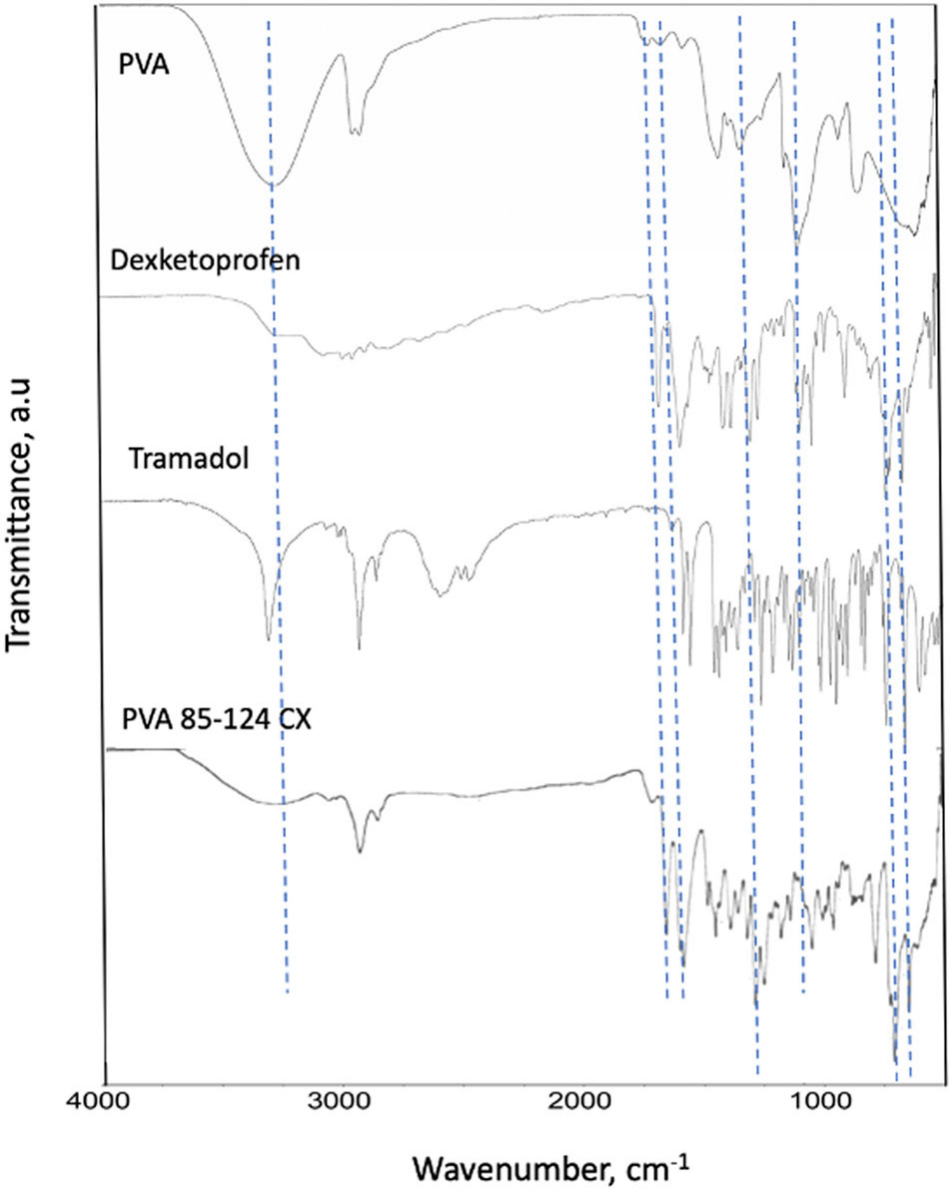
FTIR spectra of PVA and crosslinked PVA

Figure 5 shows the spectra corresponding to the raw materials, PVA, Dexketoprofen, and Tramadol, as well as a representative crosslinked PVA film after the release test. For the PVA, one can clearly observe the characteristic bands associated with the hydroxyl groups (-OH) at 3250 and 1080 cm^−1^ due to the stretching and bending vibration modes, respectively. The bands at 2940 and 2910 cm^−1^ correspond to the asymmetric and symmetric stretching of the methylene groups, and other characteristic bands at 1415, 1320, and 825 cm^−1^ correspond to CH_2_ rocking, CH wagging, and C-C stretching, respectively.

In the case of the crosslinked PVA, the intense bands at 3250 and 1080 cm^−1^ were clearly diminished, and new bands appeared (see the dotted lines in Fig. 5). For example, the new band at 1750 cm^−1^ corresponds to the carbonyl (>C=O) associated with an ester group and/or a carboxylic acid, and such a band does not appear in the pure PVA, which is indicative of the esterification reaction between PVA and citric acid. Other bands at 1250, 790, and 700 cm^−1^ that appear in the crosslinked PVA sample are associated with the presence of remaining Dexketoprofen and Tramadol.

### Swelling capacity

The swelling capacity of PVA-based films was calculated (Table 2). Crosslinked films had a lower swelling capacity than the non-crosslinked films that dissolved and were unable to continue in the test. In terms of molecular weight, samples with the lower-molecular-weight PVA (13–23) exhibited a lower swelling capacity, probably due to the low number of entanglements between polymeric chains. Samples with medium molecular weight (31–50) had the most swelling, and in the higher-molecular-weight samples (85–124), there was a reduction in swelling.

**Table 2:** Swelling capacity of PVA-based films

PVA film	*Wd* (±SD)	*Ws* (±SD)	*Te* (min)	Swelling capacity (%)
13–23	22.8 (0.05)	54.8 (1.35)	300	240.3
31–50	24.8 (0.83)	79 (0.11)	120	318.5
85–124	23.8 (0.27)	61 (2.46)	180	256.3
13–23 cx	22 (1.00)	28 (1.53)	180	127.2
31–50 cx	21.6 (0.57)	28.6 (1.01)	180	132.4
85–124 cx	22.6 (0.57)	28.3 (0.19)	180	125.2

*Wd* dry weight (mg), *Ws* weight after swelling (mg) (values were registered when films reached equilibrium swelling), *Te* time when the films reached equilibrium swelling

### In vitro drug release

**Fig. 6:**
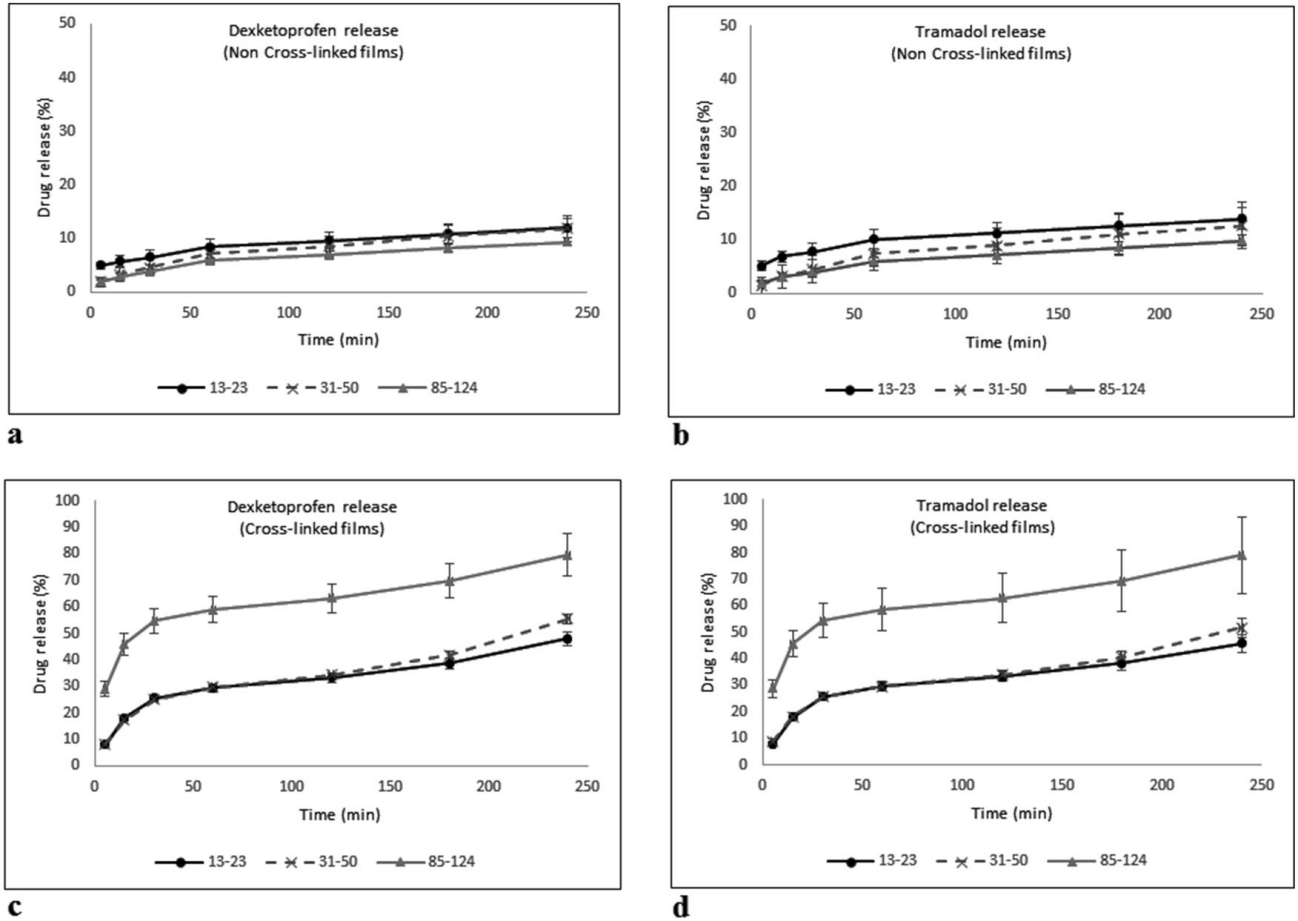
TDC cumulative release from PVA films: **a** Dexketoprofen release, non-crosslinked films; **b** Tramadol release, non-crosslinked films; **c** Dexketoprofen release, crosslinked films; and **d** Tramadol release, crosslinked films

The cumulative release (Fig. 6) showed that, in the non-crosslinked PVA films, the total release after 240 min was between 11 and 14% of the total loaded TDC. The 13–23 films showed the highest amount of drug release, and all other films had a lower TDC release. Crosslinked PVA films had a release rate of ~80% of the total TDC loaded after 240 min of testing, and the 85–124 PVA film showed similar release behavior for both Tramadol and Dexketoprofen.

Results showed a statistically significant difference (*p* < 0.05) when crosslinked and non-crosslinked PVA films were compared for both components of TDC. The results showed that the Mw of PVA exerted no interference in the kinetic release of TDC by increasing or decreasing the release time independently of the crosslinking (*p* > 0.05).

### PT and PTT

Table 3 shows the results obtained from the PT and PTT test. The statistical analysis showed statistically significant differences between experimental samples and the control for the PT (*p* < 0.05) but not for the PTT (*p* > 0.05).

**Table 3:** Prothrombin time (PT) and partial thromboplastin time (PTT) values

Sample	PT (s)	PTT (s)
Control	12.03 ± 0.12	23.17 ± 1.15
A1	13.43 ± 0.80	22.70 ± 1.93
A2	12.97 ± 0.15	21.70 ± 0.17
A3	12.93 ± 0.06	22.83 ± 0.12
B1	11.93 ± 0.21	23.07 ± 0.50
B2	12.85 ± 0.35	22.93 ± 0.84
B3	13.97 ± 0.40	23.60 ± 0.79
C1	12.60 ± 1.14	23.03 ± 0.72
C2	13.87 ± 0.25	23.57 ± 0.75
C3	11.73 ± 0.21	22.83 ± 1.00

## Discussion

The films developed in this study might favor local analgesia directly on the surgical site by releasing the TDC immediately after being placed on the wound, especially during the first hours after the surgical procedure, when the anesthetic effect decreases. The development of polymeric films loaded with TDC requires the use of water-soluble polymers with suitable film-forming properties. Polymers such as PVA have been explored in previous studies as having appropriate film-induction capabilities by the solvent casting method [16–19]. PVA was used for a cast solution in PTFE dishes, and the films obtained had different thicknesses, depending on the dimensions of the plate where the PVA films were made and on the crosslinking procedure. Many crosslinking agents are used for PVA, but citric acid was used since it is a non-cytotoxic agent that can promote crosslinking [20]. In terms of thermal characterization, the DSC results showed that crosslinking was achieved because the Tg values for the PVA were changed as a consequence of such a reaction. The Tg indicates the coordinated movement of several polymeric molecules and is associated with flexibility due to the amorphous nature of a polymer. When the polymers are partially crosslinked, a less flexible polymeric structure is generated, and higher energy is required to promote the coordinated movement of the chains; therefore, the Tg value is displaced to higher values, as occurred in our case, and as can be observed in Fig. 4.

The crosslinking reaction between PVA and citric acid has been well-confirmed by fourier transform infrared spectroscopy (FTIR) by do Nascimento et al., who observed that the higher the concentration of citric acid, the higher the crosslinking reaction [21]. In our study, we analyzed our samples by FTIR, before and after the crosslinking reaction. Figure 5 clearly shows that a new band associated with the carbonyl group from ester groups appears. Esterification can take place when the carboxylic acid groups from citric acid react with the hydroxyl groups from the PVA. Such a reaction creates a 3D crosslinked structure, since the molecules of citric acid can react with different polymeric chains of PVA, as shown.

In addition, it can be seen (dotted lines in Fig. 5) that the TDC used remained in the crosslinked films. As will be discussed later, most, but not 100%, of the TDC was released, and some characteristic bands appeared in the crosslinked film after the release test. This could be due to the network structure generated by the crosslinking reaction, which could trap some TDC molecules.

SEM images showed the presence of particles on the surface that could be a result of incomplete dissolution of PVA in the non-crosslinked films. Moreover, these films had a porous surface with a heterogeneous pore size on all the films, independent of PVA molecular weight. Conversely, crosslinked PVA films had a nonporous surface without particles, which could result from the formation of a highly dense polymeric network, probably formed due to the significant entanglement of polymeric chains or hydrogen bonding between them, reducing the availability of the functional groups to interact with water molecules [22].

**Fig. 7:**
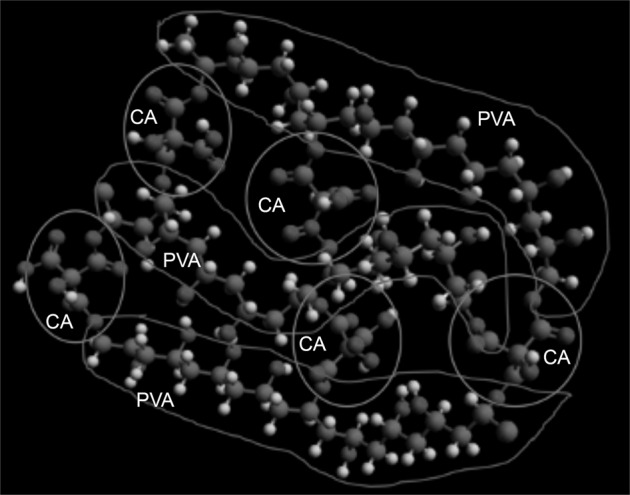
Proposed crosslinked structure of PVA by citric acid. PVA chains are enclosed and identified with “PVA” and citric acid is enclosed by a circle and identified by “CA”

Swelling capacity was controlled by the addition of a crosslinker, indicating diminished swelling due to a poorly crosslinked system [23]. The decreased swelling capacity was related to an increase in drug release rate. This behavior could be a result of the interaction between the citric acid and OH groups of PVA, leaving molecules of the TDC to remain free and to be more easily released [23]. Moreover, the dissolution started within the first 24 h in the presence of citric acid, compared with non-crosslinked films, where the dissolution started between 12 and 48 h. Dissolution was slower in films synthesized with high-molecular-weight PVA, but also in this case the release rate was faster. TDC release was significantly different between crosslinked and non-crosslinked PVA films, independent of PVA molecular weight. On the basis of the formulation of the PVA films, we calculated a theoretical loading of 2000 μg for Tramadol and 1350 μg for Dexketoprofen in the 2-mg samples that we used for testing drug release. Around 30% of TDC was released from PVA films 85–124 (~600 μg Tramadol and 400 μg Dexketoprofen) at 5 min of evaluation as an initial burst release. After that period, TDC release increased up to 60% after ~50 min of evaluation and continued increasing at a sustained rate until reaching around 80% at 240 min of testing. In the present results, it was observed that the release of TDC from the films showed a burst release in the initial times of evaluation and a sustained release in the subsequent periods of the test. These results suggest that an esterification reaction occurred between citric acid and PVA, leaving the drug molecules free to be released into the aqueous medium. Other authors [24] have confirmed the esterification between citric acid and PVA using proportions similar to those used in our study and have concluded that esterification through a polycondensation reaction leads to a material with adequate properties to be used as a scaffold for body tissues. Rocha-García et al. [25] used citric acid as a crosslinker to create poly(ethylene glycol)-diamine hydrogels for drug release in vitro, with Tramadol as a model drug. They proposed a mechanism of physical crosslinking and showed a release profile that depended on the presence of citric acid. Anepu et al. [26] evaluated the interaction between Tramadol and different polymers used to formulate gastro-retentive floating tablets. They confirmed, by FTIR analysis, that there were no drug interactions. In our study, the films crosslinked with citric acid had a higher release in comparison with the non-crosslinked PVA films; moreover, crosslinked films 85–124 had the highest release when compared with crosslinked films 13–23 and 31–50. This can be attributed to a better 3D network formed with the crosslinking along with the entanglements, which were more numerous as the molecular weight increased (Fig. 7).

Deionized water (pH 7) was selected as the release reservoir, since it was considered the most inert solution for this stage, especially since this experiment was not designed to evaluate a single molecule but rather a pharmacological combination. The use of a buffer solution may affect (positively or negatively) the release of the drugs and, consequently, the behavior of the films. However, further experiments are already being designed to evaluate the behavior of these films in different media such as buffer solutions and on different pH levels, or even in biological fluids such as blood or saliva. A previous study [27] reported that a citric acid crosslinked film performed with carboxymethyl cellulose for controlled release of ketoconazole and confirmed the use of citric acid as an effective crosslinking agent in controlled-release devices. In our study, we proposed a local release directly at the site of intervention, with doses in the microgram range, to obtain local direct multimodal analgesia with TDC [7], thus reducing systemic side effects. A study that evaluated the use of porous materials to control the release of analgesics [2] reported a release rate of 77% Dexketoprofen after 300 min, which differs from our results obtained from non-crosslinked PVA films, which, after 240 min, had a release rate between 5 and 15%. Crosslinked PVA films showed a TDC release around 80% of the total drug loaded after 240 min in the case of 85–124 PVA film as a result of citric acid´s crosslinking effect, promoting a sustained release of TDC.

Regarding the lack of porosity of crosslinked PVA films, it was shown that this factor did not affect the release rate. Compared with reports of porous materials [2, 28], the obtained crosslinked PVA films showed a sustained release for 240 min. Total TDC released from crosslinked PVA films showed an efficient release rate compared with other matrix materials (such as natural gums used to make tablets) [29]. Such behavior offers a suitable option for the management of local acute postoperative analgesia.

The composition of the films is a factor that plays an important role in the controlled release of the drugs. In the case of our films, the simple mixture of PVA and TDC and the crosslinking with citric acid led to material with an optimal release rate and an adequate dissolution compared with those of other materials tested and designed for controlled release [30]. PVA films obtained through the solvent casting method in this study can be mixed with other drugs and different polymers to synthesize drug-release vehicles that could be used to treat complete wounds [31], although more experiments are necessary to develop a new material with these properties.

Normal PT and PTT values are 11.1–14.3 and 24–36 s, respectively, according to the assay that we performed for this study [14, 15]. The PVA films induced PT values that showed statistically significant differences in comparison with the PT value observed in the control, but those values were in the normal range. Hence, the PVA films did not negatively prolong the PT. The PVA induced values of PTT that were also in the normal range. Taking into account the intended use of the PVA films in tissue after surgical procedures, the in vitro observations allow for expectations that the PVA will not delay blood coagulation. PVA is a non-ionic polymer whose characteristics help to reduce interaction with the proteins of the blood coagulation pathways [15]. Future studies should be performed to test the in vivo effects of the PVA films.

One limitation of this study was a drug release time of only 4 h. The non-crosslinked PVA films and the lowest-Mw PVA films used here underwent an important physical change at 240 min because of prolonged contact with the deionized water, and the films were difficult to handle for the drug-release experiments. Some of those samples suffered depletion. Hence, to achieve the same evaluation time for all the films, we stopped the drug release evaluation at 240 min. A longer release time allows for a longer evaluation time, which can provide valuable evidence to establish the approach of local medication to control PP as a common clinical practice. The lack of data from characterization at this stage of the project could be considered as a second limitation, but further studies must validate this alternative as a viable approach to control release in vitro and in vivo and to evaluate its effect.

## Conclusions

The results demonstrate that it was possible to design a PVA film crosslinked with citric acid and loaded with TDC. The use of citric acid as a crosslinking agent had a positive effect on drug release, promoting a more effective release rate of both drugs. The molecular weight of PVA tested here did not affect the kinetic release of TDC.

## Supplementary information

Supplementary Information
