# Supplementary material for: Synthesis of a PVA drug delivery system for controlled release of a Tramadol–Dexketoprofen combination
Source: J Mater Sci Mater Med. 2021 May 7;32(5):56. doi: 10.1007/s10856-021-06529-3 (PMC8105240; doi:10.1007/s10856-021-06529-3)
Supplement: Supplementary file 1 — Supplementary Information [file 10856_2021_6529_MOESM1_ESM.docx]

**Organoleptic properties of PVA-based films loaded with TDC. Thickness is expressed as the mean (mm) and standard deviation (SD).**

|  | 13-23 | 13-23 cx | 31-50 | 31-50 cx | 85-124 | 85-124 cx |
| --- | --- | --- | --- | --- | --- | --- |
| Color | Light yellowish | Yellowish | White | Yellowish | White | Brown-yellowish |
| Transparency | + | ++ | + | ++ | + | ++ |
| Gloss | --- | + | --- | + | --- | + |
| Flexibility | +++ | -- | +++ | -- | +++ | - |
| Elasticity | ++ | -- | ++ | -- | ++ | -- |
| Integrity | +++ | ++ | +++ | + | +++ | ++ |
| Smoothness | +++ | +++ | ++ | +++ | + | +++ |
| Thickness | 2.02 (0.0019) | 0.29 (0.010) | 2.00 (0.008) | 0.29 (0.010) | 2.1 (0.80) | 0.29 (0.009) |
